# Reversibility of Aβ oligomer neurotoxicity

**DOI:** 10.18632/oncotarget.19083

**Published:** 2017-07-07

**Authors:** Wataru Araki

**Affiliations:** Department of Demyelinating Disease and Aging, National Institute of Neuroscience, National Center of Neurology and Psychiatry, Tokyo, Japan

**Keywords:** Alzheimer’s disease, amyloid β-protein, neuron, neurotoxicity, oligomer

Patients with Alzheimer’s disease (AD), the most common neurodegenerative dementia disorder, chiefly suffer from impairment of memory and other cognitive functions. AD is neuropathologically characterized by senile plaques and neurofibrillary tangles, which are composed of amyloid β-protein (Aβ) and phosphorylated tau proteins, respectively. Recently, a new concept has emerged: that soluble oligomeric forms of Aβ (Aβ oligomers), but not Aβ fibrils, play a primary pathogenic role in the pathological cascade of AD [1, 2]. This idea is based on findings that soluble forms of Aβ provoke neurotoxic effects, including tau abnormalities (especially hyperphosphorylation), functional and structural abnormalities of synapses, and induction of neuronal death. This concept is supported by numerous studies that have employed a variety of experimental systems, including cell culture, brain slices and animal models, as well as the fact that Aβ oligomers are abundant in post-mortem AD brains [1, 2]. Thus, oligomeric Aβ is considered a major culprit in the molecular pathology of AD.

**Figure 1:**
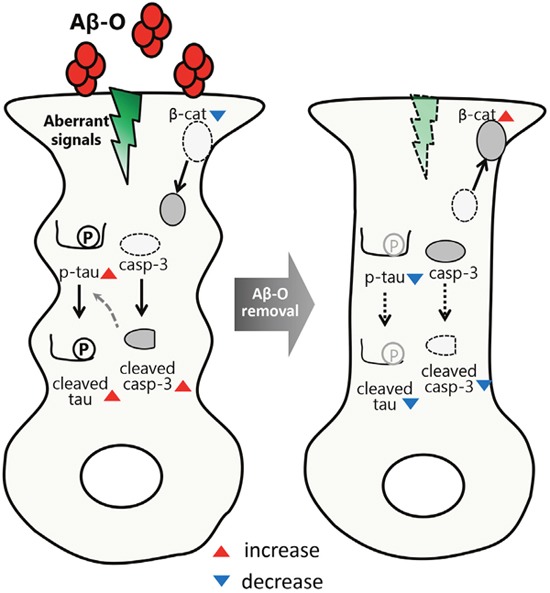
Reversibility of Aβ oligomer neurotoxicity Casp-3: caspase-3; β-cat: β-catenin; p-tau: phosphorylated tau.

To investigate the pathological roles of Aβ oligomers, we have established a neuron culture model system, in which rat primary neurons are exposed to relatively low concentrations (~2.5 μM) of Aβ42 oligomers (Aβ-O) for relatively long periods (2-3 days) [3, 4]. We observed that Aβ-O induces neurotoxic insults with limited cell death under these conditions, producing effects that include activation of caspase-3 and eIF2α (eukaryotic translation initiation factor 2α), indicative of induction of apoptosis and other stress responses; abnormal alterations of tau proteins (increased phosphorylation and caspase-mediated cleavage); as well as abnormal alterations of β-catenin (reduced protein levels and dislocalization) (Figure 1) [3, 4]. β-catenin is known to play important roles in regulating synaptic structures and plasticity as well as Wnt signaling [5]. Because these changes are reflective of characteristic pathological features of AD, this neuron model is considered a useful system for investigating the neurotoxic mechanisms triggered by Aβ oligomers.

We were interested in the question of whether the neurotoxicity of Aβ oligomers is reversible and abates upon their removal, an issue that has remained largely unexplored. To investigate this possibility, we designed the following experimental paradigm: Rat primary cultured neurons were treated with Aβ-O for 2 days, at which point cells were deprived of Aβ-O by replacing the medium with fresh medium lacking Aβ-O, or were re-provided Aβ-O, and cultured for an additional 2 days; untreated neurons were used as controls. Neurons continuously treated with Aβ-O showed greater activation of caspase-3 and eIF2α, and exhibited persistent, abnormal alterations of tau and β-catenin. In contrast, upon Aβ deprivation, caspase-3 and eIF2α activation were considerably attenuated, aberrant phosphorylation and caspase-mediated cleavage of tau recovered for the most part, and abnormal alterations of β-catenin were partially reversed (Figure 1). Notably, Aβ-O-induced β-catenin dislocation appeared to be associated with perturbation of synaptic organization [4]. These results indicate that removal of extracellular Aβ-O can fully or partially reverse Aβ-O-induced neurotoxic and synaptotoxic alterations in our neuron model. Our findings suggest that Aβ oligomer-associated neurotoxicity is a reversible process in that neurons are capable of recovering from moderate neurotoxic insults. These data also support the idea that Aβ oligomers act on the cell surface of neurons to transmit aberrant signals, resulting in various abnormal cellular responses; upon Aβ oligomer removal, the aberrant signals subside, resulting in reversal of all abnormal responses (Figure 1).

A few previous studies have obtained results consistent with the reversible toxicity of Aβ oligomers. For example, a study using mouse organotypic slices found that Aβ-induced spine loss recovers following Aβ washout [6]. However, several important issues have yet to be elucidated. Oligomeric Aβ possibly exerts its effect through Aβ oligomer receptors on the cell surface; however, which receptors or molecules among a variety of candidates are genuine mediators of Aβ oligomer toxicity remains uncertain [2]. How Aβ oligomers almost simultaneously affect synapses, tau, and cell-death pathways is also an important question for further investigation. Additionally, it remains unclear which types of Aβ oligomers among the various forms with different molecular sizes are most bioactive [2, 7].

The reversible nature of the neurotoxicity of Aβ oligomers has significant implications for therapeutic strategies for AD. Notably, any treatment designed to remove or reduce Aβ oligomers could be effective in halting or even reversing the progression of the very early pathology of AD. Currently, immunotherapy targeting Aβ oligomers and inhibitors of BACE1 (an essential protease for Aβ production) are promising therapeutic options [7, 8]. Alternatively, any agent that protects neurons from Aβ oligomer neurotoxicity may be beneficial as a complementary treatment. It should be possible to verify the clinical efficacy of drugs targeting Aβ oligomers in future clinical trials in which individuals with mild cognitive impairment (prodromal AD) or preclinical AD are enrolled.
